# Anomaly Detection in Annular Metal Turning Surfaces Based on a Priori Information and a Multi-Scale Self-Referencing Template

**DOI:** 10.3390/s23156807

**Published:** 2023-07-30

**Authors:** Xinyu Suo, Jie Zhang, Jian Liu, Dezhi Yang, Feitao Zhou

**Affiliations:** State Key Laboratory of Advanced Design and Manufacture for Vehicle Body, Hunan University, Changsha 410082, China; suoxinyu@hnu.edu.cn (X.S.); zhangjie@hnu.edu.cn (J.Z.); yangdezhi@hnu.edu.cn (D.Y.); feitaozhou@hnu.edu.cn (F.Z.)

**Keywords:** image anomaly detection, annular workpiece, a priori and self-information

## Abstract

To solve the problem of anomaly detection in annular metal turning surfaces, this paper develops an anomaly detection algorithm based on a priori information and a multi-scale self-referencing template by combining the imaging characteristics of annular workpieces. First, the annular metal turning surface is unfolded into a rectangular expanded image using bilinear interpolation to facilitate subsequent algorithm development. Second, the grayscale information from the positive samples is used to obtain the a priori information, and a multi-scale self-referencing template method is used to obtain its own multi-scale information. Then, the phase error and large-size anomaly interference problems of the self-referencing method are overcome by combining the a priori information with its own information, and an accurate response to anomalous regions of various sizes is realized. Finally, the segmentation completeness of the anomalous region is improved by utilizing the region growing method. The experimental results show that the proposed method achieves a mean *pixel AUROC* of 0.977, and the mean *M_IOU* of segmentation reaches 0.788. In terms of efficiency, this method is also much more efficient than the commonly used anomaly detection algorithms. The proposed method can achieve rapid and accurate detection of defects in annular metal turning surfaces and has good industrial application value.

## 1. Introduction

There are a lot of grooves, tapered surfaces, end faces and other annular turning surfaces in the manufacturing process of metal workpieces [1,2,3]. Surface anomaly detection in annular metal turning surfaces is an important process for the quality control of machining. In images, the annular metal turning surface is a circular structure, and its region of interest occupies only a small part of the whole image [4]. In grayscale images, the annular metal turning surface shows a uniform distribution of grayscale in the circumferential direction. Developing a general and efficient anomaly detection algorithm using the structure and grayscale characteristics of annular metal turning surfaces has important application value for automated anomaly detection in annular metal turning surfaces.

Two problems need to be overcome to achieve anomaly detection in annular metal turning surfaces: 1. large image resolution but small region of interest; 2. metal turning surface positive and negative samples are unbalanced, and negative samples are difficult to collect.

In an image containing a complete annular metal turning surface, the image size is determined by the outer diameter of the ring. The difference between the inner and outer diameter of the ring is usually not large, which results in the region of interest of the annulus occupying a small area in the image. Regarding the problem of high-resolution anomaly detection, in addition to the direct processing of the whole image [5,6], other common solutions are resizing [7] and cutting [8,9]. Zhao [10] et al. directly compressed the input image size, and experiments proved that such a strategy has little effect on the detection accuracy for large-size targets, but the recognition effect is poor for small-size targets, and the direct resize neutralizes or removes the pixels in small target regions. Some researchers have used a cutting method to cut large-size images into small-size images for processing and finally stitch the detection results together as the final output. The sliding window approach [11,12,13] has also been adopted by many researchers to transform the problem of detecting high-resolution images into a small sliding window detection problem. Coordinate transformation is also an effective way to extract the ring region of interest. Suo [4] and Deng [14] et al. directly unfolded the region of interest of annular workpieces to complete the inspection of the annular workpieces without reducing the resolution of the part being inspected. The conversion of the annular region into a rectangular region also facilitates subsequent algorithm development.

In terms of industrial anomaly detection needs, the collection of anomaly samples is more difficult due to the low product substandard rate. Meanwhile, anomalies have different manifestations, and it is difficult to cover all types of anomalies in the collection of anomaly samples, and unexpected anomalies are usually encountered in actual production. The difficulty of collecting negative samples leads to the difficulty of practical application of supervised target retrieval [15,16] or segmentation [17,18] methods. To address the problem of negative samples being difficult to collect, researchers have carried out extensive research work on positive-sample-based anomaly detection algorithms. Traditional anomaly detection algorithms can quickly achieve anomaly detection by manually designing the processing flow and using morphological processing [19,20] or template matching [21,22]. However, the traditional algorithm has high requirements for illumination, imaging angle and the state of the workpiece to be detected, and the selection of each parameter in the algorithm is complicated, and the algorithm is less robust. With the development of deep learning technology, the powerful feature extraction capability of deep networks has greatly enriched the technical methods of unsupervised anomaly detection. Positive sample anomaly detection methods using deep learning networks can be classified into two main categories [23]: image-reconstruction-based methods [24,25,26] and discriminative-embedding-based methods [27,28]. Image-reconstruction-based methods tend to learn the structural information of positive samples for image reconstruction and compare the reconstruction differences between the image to be detected and the abnormality-free map to achieve abnormality detection. Discriminative-embedding-based methods are more about learning the feature distribution of positive samples, and the determination of anomalous regions is achieved by calculating the distance between the features of the samples to be detected and the statistical positive sample features. Defard et al. [29] used VGG16 as a feature extraction layer, patch embedding for anomaly-free samples using multivariate Gaussian distribution to obtain a probabilistic representation of the normal class, and anomaly detection was achieved by calculating the Mahalanobis distance between the image to be detected and the normal class. Roth et al. [30] combined embeddings from ImageNet models with an outlier detection model to construct a maximally representative memory bank of nominal patch features. This method provides a further improvement in the speed of anomaly detection. The detection effect of deep-learning-based anomaly detection methods depends mainly on the learning effect of the network, and it is more difficult to improve the targeted effect of the trained network. Additionally, anomaly detection by deep learning methods generally results in too-large segmentation of small-sized anomalous regions.

Since the annular metal turning surface has a uniform grayscale in the circumferential direction, this grayscale characteristic is suitable for the self-referencing template method in the traditional anomaly detection method. The self-referencing method uses the periodic grayscale information of the image itself to construct an anomaly-free template, and the final anomaly detection result is obtained by the difference between the template and the image to be detected [31,32]. Ideally, the self-referencing template is anomaly free, but when there are large-size anomalous regions, the self-referencing template constructed using its own grayscale information contains a large amount of anomalous information, which causes serious misidentification problems [33]. Due to the use of grayscale differencing, the self-referencing template method may be incomplete in detecting anomalous regions with low contrast.

Combining the above analysis and the existing problems, this paper combines the circular structure of the annular metal surface and the characteristics of uniform imaging to unfold the annular region of interest, which not only maintains the pixel resolution of the region of interest but also reduces the amount of data processing and the background complexity of anomaly detection objects. Due to the lack of anomalous samples, this paper proposes to implement anomaly detection in annular metal surfaces based on a self-referencing algorithm. In this paper, we overcome the problem of the self-referencing template method failing to detect large-size anomalous regions by introducing a priori information and introduce the strategy of a multi-scale self-referencing template and region growth to improve the accuracy of low-contrast anomaly detection.

Our main contributions in this article are summarized as follows:

(1) A bilinear interpolation expansion method based on the principle of transformation between polar and Cartesian coordinate systems is invoked for the problem of extracting the region of interest of annular metal turning surfaces;
(2) For the problem of large-size anomaly detection, a general self-referencing template anomaly detection algorithm that fuses a priori information and its own grayscale information is proposed, and the anomaly segmentation effect is further improved by using a region growing method;
(3) A more systematic and comprehensive experimental analysis and demonstration of the selection basis and performance effect of the parameters in the algorithm of this paper are conducted, and the generality of the parameters is proved through experiments.

The remainder of this paper is organized as follows. The flow of the developed algorithm and the object to be tested are briefly introduced in Section 2. Section 3 details the implementation of the entire algorithm. The following Section 4 describes the experimental design and results. Finally, this work is concluded in Section 5.

## 2. Pipelines and Sample Acquisition

### 2.1. Pipeline

**Figure 1:**
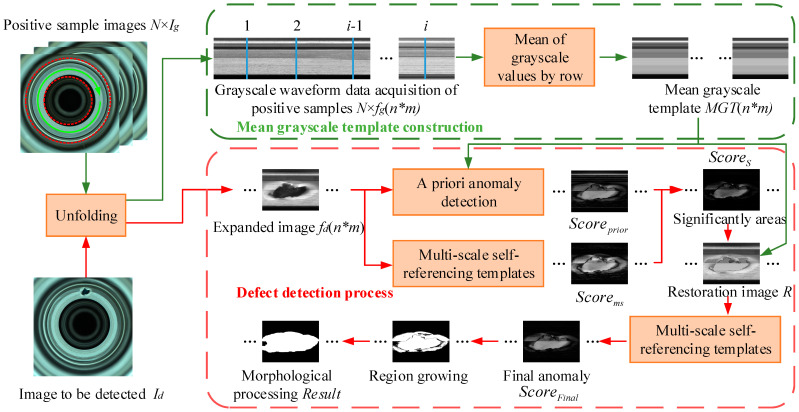
Pipeline of anomaly detection algorithms based on a priori information and multi-scale self-referencing template.

The process of manual anomaly detection is: first, find the region of interest; then, judge the rough anomaly region based on the a priori information in the mind; finally, finish the precise location of the anomaly region based on the information relating to texture or the grayscale of the image itself. Referring to the process of manual anomaly detection, this paper carries out the development of an anomaly detection algorithm for annular metal turning surfaces. The pipeline of the proposed method is illustrated in Figure 1, which is mainly divided into two parts: mean grayscale template construction and the defect detection process. Firstly, the annular region of interest (ROI) in positive samples $I_{g}$ is unfolded into rectangular image $f_{g}$ (corresponding to the manual detection of the search of interest).

In the mean grayscale template construction part, the grayscale value data of i columns in N normal expanded images are randomly extracted, and a total of N × i columns of waveform data are extracted, and the grayscale mean value of each row is calculated as the standard grayscale waveform to construct mean grayscale template MGT with the same size as the input image (corresponding to the a priori information of manual detection). In the defect detection process, for the expanded image to be detected $f_{d}$, the significant anomalous regions are screened using the mean grayscale template and the multi-scale self-referencing template (corresponding to the coarse anomaly detection for manual detection); the significant anomalous regions are restored using MGT data to obtain the restoration map R; again, using the multi-scale self-referencing template method to obtain the final anomaly difference score map ${Score}_{final}$, the high-response region in ${Score}_{final}$ is the anomaly region (corresponding to the precise location of the manually detected anomaly region); finally, the abnormal region is segmented by the region growth and morphology processing method, and the segmentation map Result is obtained.

### 2.2. Sample Acquisition

**Figure 2:**
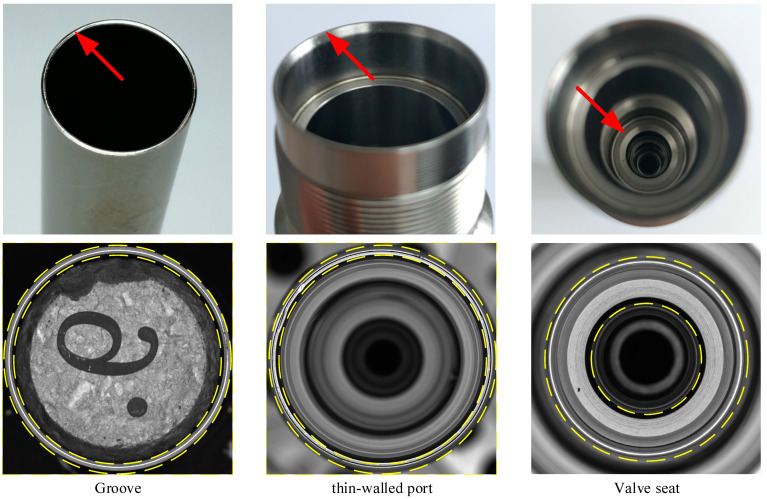
Annular metal turning surface dataset.

Since there are relatively few public datasets for anomaly detection in annular metal turning surfaces, in this paper, as shown in Figure 2, a dataset containing three types of annular metal turning surfaces is constructed: a nuclear fuel rod groove dataset, rotary body thin-walled port dataset, and injector nozzle valve seat surface dataset. The objects to be detected in the datasets are all annular workpieces (yellow area), and their unfolded images are all long-bar images; the nuclear fuel rod groove has an unfolded size of 100 × 7000 pixels, the rotary thin-walled port has an unfolded size of 120 × 8700 pixels and the injector valve seat surface has an unfolded size of 440 × 10,000 pixels. The number of positive and negative samples for the three datasets is shown in Table 1.

## 3. Algorithm Implementation

### 3.1. Unfolding of Annular Region of Interest

The region of interest of the annular turning surface only occupies part of the whole image. To reduce the number of data operations and make full use of the structural characteristics of the annular surface with uniform grayness in the circumferential direction, the annular region of interest needs to be extracted and unfolded into a long rectangular image.

**Figure 3:**
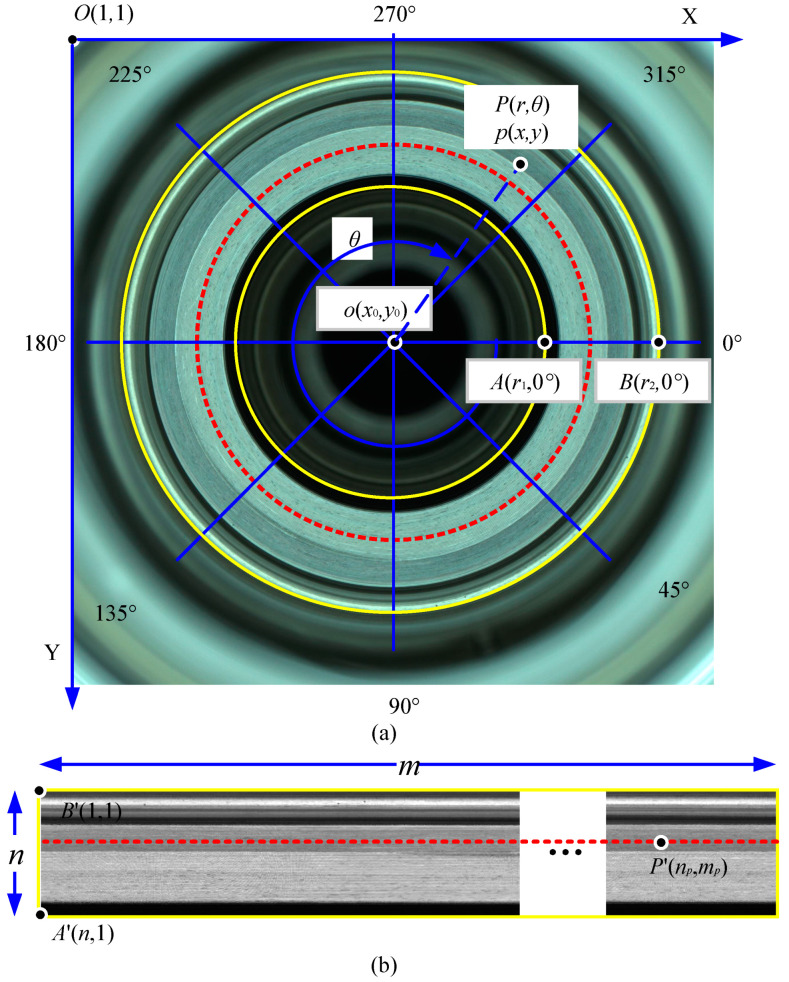
Annular region of interest unfolding. (**a**) The image Cartesian and polar coordinate system I(x,y); (**b**) expanded image f(x,y).

In Figure 3, the schematic diagram of the annular region of interest unfolding is shown. The yellow area in Figure 3a is the annular region of interest, and its corresponding annular unfolding area is shown in Figure 3b. To facilitate the annular unfolding operation, two coordinate systems are constructed in the original image I(x,y): the image Cartesian coordinate system O−XY and the polar coordinate system o−rθ with the center of the annular region of interest as the origin. Let P(r,θ) be any point of the annular region in polar coordinates, and its corresponding point in the image Cartesian coordinate system is p(x,y), and the relationship between the image Cartesian coordinate system and the polar coordinate system can be established.

(1) $$\left\{\begin{array}{cc} \begin{array}{c} x=x_{0}+r\cos\theta \\ y=y_{0}+r\sin\theta \end{array} & \theta \in [0{}^{\circ},90{}^{\circ})\\ \begin{array}{c} x=x_{0}-r\cos\theta \\ y=y_{0}-r\sin\theta \end{array} & \theta \in [90{}^{\circ},180{}^{\circ})\\ \begin{array}{c} x=x_{0}+r\cos\theta \\ y=y_{0}+r\sin\theta \end{array} & \theta \in [180{}^{\circ},270{}^{\circ})\\ \begin{array}{c} x=x_{0}-r\cos\theta \\ y=y_{0}-r\sin\theta \end{array} & \theta \in [270{}^{\circ},360{}^{\circ}) \end{array}\right.$$

where $x_{0}$ and $y_{0}$ are the coordinates of the polar coordinate system o−rθ in the center circle of the image coordinate system.

The inner and outer rings of the annular region of interest intersect the 0° axis of the polar coordinate system at the points $A(r_{1},0{}^{\circ})$ and $B(r_{2},0{}^{\circ})$, whose corresponding positions in the expanded image f(x,y) are $A^{\prime }$ and $B^{\prime }$, respectively. Since the circumferences of the circles where points A and B are located are different, in this paper, the annular region of interest is unfolded along the circumference of the outer ring. Let the image f(x,y) size be n × m after the annular unfolding, which is defined as follows:

(2) $$\begin{array}{l} m=\left\lfloor 2\pi r_{2}\right\rfloor \\ n=r_{2}-r_{1}+1 \end{array}$$

where n is the number of rows of the expanded image, and m is the number of columns of the expanded image; $r_{1}$ and $r_{2}$ are the radius of the inner and outer rings, respectively, and their values are integers; and ⌊ ⌋ is a downward rounding symbol.

Let the point P(r,θ) in the polar coordinates have its corresponding position as $P^{\prime }(n_{p},m_{p})$ in the expanded image. Then, the transformation equation of point P to $P^{\prime }$ is:

(3) $$\left\{\begin{array}{l} n_{p}=\left\lfloor r-r_{1}\right\rfloor \\ \theta =\frac{360{}^{\circ}}{m}(m_{p}-1) \end{array}\right.\to \left\{\begin{array}{l} n_{p}=\left\lfloor r-r_{1}\right\rfloor \\ m_{p}=\left\lfloor \frac{m\theta }{360{}^{\circ}}+1\right\rfloor \end{array}\right.$$

where $n_{p}$ and $m_{p}$ are the coordinates of point $P^{\prime }$ in the expanded image; m is the number of columns of the expanded image; r is the radius coordinate of point P in the polar coordinate system $r=r_{1},r_{1}+1,\ldots ,r_{2}$; θ is the angular coordinate of point P in the polar coordinate system $\theta =\frac{360{}^{\circ}}{m}(t-1),t=1,2,\ldots ,m$; and ⌊ ⌋ is a downward rounding symbol.

**Figure 4:**
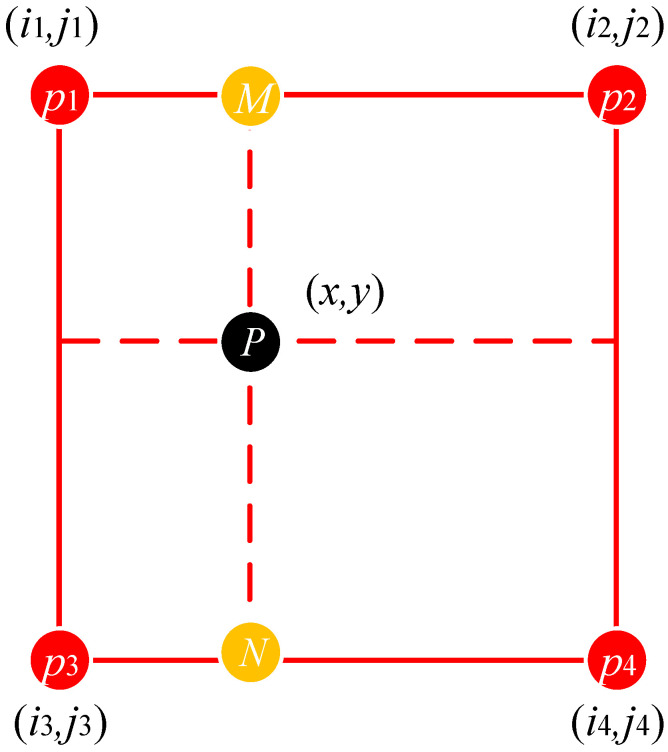
Bilinear interpolation method.

To improve the unfolding effect of the annular turning surface and avoid the jagged effect generated by the image unfolding, the gray values in the unfolding map are calculated by bilinear interpolation when performing the unfolding. As shown in Figure 4, p(x,y) is the interpolation point corresponding to point $P^{\prime }(n_{p},m_{p})$ in the expanded image. Since the coordinates of p(x,y) may not be an integer, four integer points $p_{1}$, $p_{2}$, $p_{3}$ and $p_{4}$ around p are selected. The values of point M and point N are obtained by linear interpolation using $p_{1}$ and $p_{2}$ and $p_{3}$ and $p_{4}$, respectively. Then, the grayscale value of point p is obtained by the linear interpolation of point M and point N.

(4) $$\begin{array}{l} p_{m}=p_{2}-(p_{2}-p_{1})(i_{2}-x)\\ p_{n}=p_{4}-(p_{4}-p_{3})(i_{4}-x)\\ p=p_{n}-(p_{n}-p_{m})(j_{n}-y) \end{array}$$

This completes the extraction and unfolding process of the annular region of interest. For any annular workpieces, we only need to determine the circle center $o(x_{0},y_{0})$, inner ring radius $r_{1}$ (in pixels) and outer ring radius $r_{2}$ (in pixels) and use the above Equations (1)–(4) in turn to achieve the counterclockwise unfolding process of the annular workpiece region of interest.

### 3.2. Mean Grayscale Template Construction

**Figure 5:**
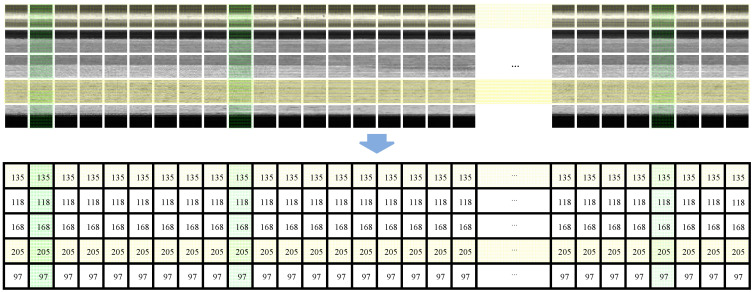
Image features of the annular expanded image.

For computational convenience, the images are converted to single-channel grayscale maps with grayscale values ranging from 0 to 1 during the subsequent algorithm development. As shown in Figure 5, for the expanded image f(x,y) of an annular workpiece without anomalies, it can be ideally considered to have equal gray values in each row (yellow region) and the same gray waveform in each column (green region).

In the actual industrial detection process, the design of the tooling fixture and imaging method can ensure that the imaging position of the annular workpiece is relatively fixed, and the imaging brightness and contrast of each position are relatively consistent, so it can be approximated that the distribution of the gray value of each row of the corresponding expanded image conforms to the normal distribution. For any annular turning surface, the mean value of each row of grayscale can be counted to construct an anomaly-free mean grayscale template MGT equal to the size of the expanded image.

N anomaly-free expanded images $I_{g}$ are collected, and a positive-sample statistical matrix Ps of size n × (N∗k) is constructed by randomly selecting k columns of grayscale waveforms in each expanded image. The mean grayscale value of each row of Ps is calculated, and the mean grayscale template MGT is constructed.

(5) $$MGP(i,:)=\frac{1}{N*k}\sum_{j=1}^{N*k}Ps(i,j)$$

where MGT(i,:) is all the grayscale values in row i of the mean grayscale template; i is the row coordinates of Ps in the range of 1,2,⋯,n; and j is the column coordinate of Ps in the range of 1,2,⋯,N∗k.

If the MGT is directly used as a template to calculate the absolute value of the difference between MGT and the image f to be detected, the a priori anomaly score ${Score}_{prior}$ can be obtained.

(6) $${Score}_{prior}=abs(MGT-f)$$

**Figure 6:**
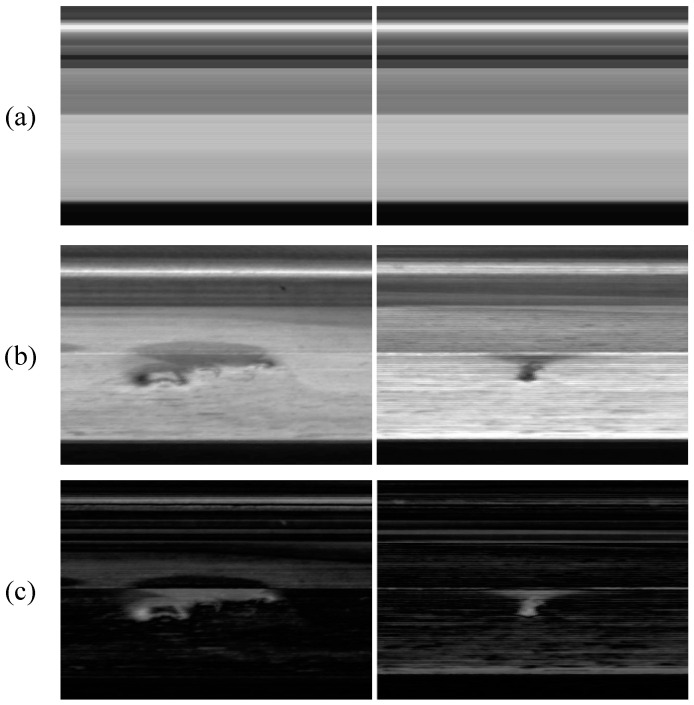
Results of the a priori anomaly scores. (**a**) Mean grayscale template MGT. (**b**) Image to be detected. (**c**) The a priori anomaly score $Score_{prior}$.

As shown in Figure 6, the results of the a priori anomaly scores for the multiple images to be detected are shown. In Figure 6c, the a priori anomaly score has a strong response for anomalous regions, but the score map also has a high response for edges and contours. The reason for this is that the MGT template can only respond to the grayscale distribution trend of the image and cannot accurately respond to the phase shift of the image. Therefore, the a priori anomaly score cannot be directly applied to the anomaly detection task.

### 3.3. Anomaly Detection Algorithm Based on a Priori Information and Multi-Scale Self-Referencing Template

**Figure 7:**
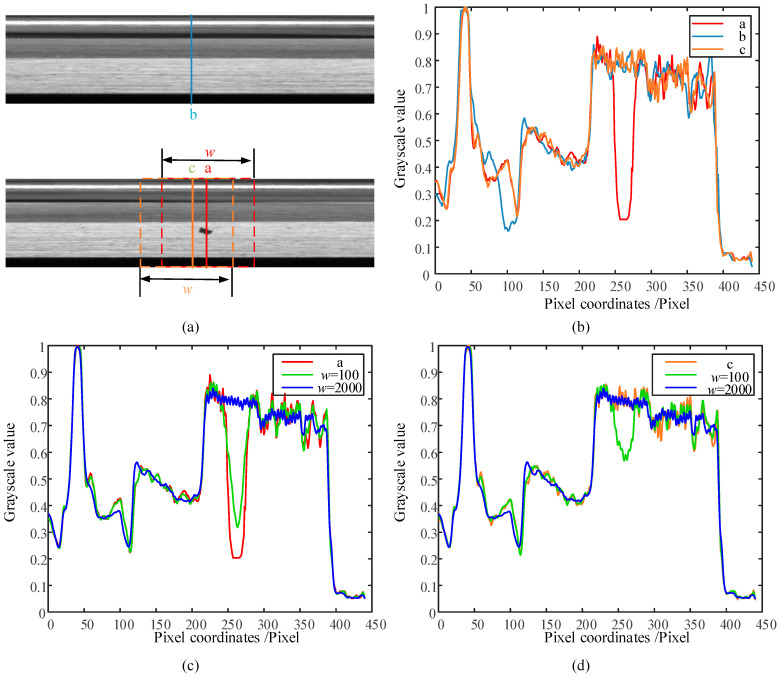
Grayscale waveform analysis of the expanded image. (**a**) Grayscale waveform selection; (**b**) grayscale waveforms of different columns; (**c**) self-referencing template waveforms of abnormal column; (**d**) self-referencing template waveforms of normal column.

In practical imaging of annular turning surfaces, there are problems such as oil interference, surface texture, uneven lighting or unfolded center shift. As shown in Figure 7b, the grayscale of each row of the expanded image is the same; meanwhile, the grayscale waveforms of each column also produce phase deviation. The dashed box in Figure 7a represents the self-reference template construction area at the solid line, and the solid line is the center of the dashed box. The construction region of the self-referencing template has a total of w columns, and the row grayscale mean of the region is used as the grayscale template. Figure 7c shows the self-referencing template waveforms of an abnormal column, where the difference at the anomaly increases as the value of w is taken, but the disturbance in other regions increases. Figure 7d shows the self-referencing template waveforms of a normal column. When w is small, there is a large amplitude difference between the constructed template and the normal waveform due to the influence of the anomalous region, and false recognition occurs at this time. When w takes the value of 2000, the amplitude of the template waveform is the same as that of the anomaly-free waveform.

The above analysis shows that, in the self-referencing template algorithm, the parameter w directly determines the performance of the algorithm. Too small a w value can create problems with false recognition and missed recognition, while too large a w value can introduce too much interference. To solve the above problems, with reference to the process of manual judgment of abnormal areas, this paper proposes a highly robust anomaly detection algorithm adapted to annular metal turning surfaces by combining abnormality-free mean grayscale templates (a priori knowledge) and a multi-scale self-referencing template (own grayscale information).

First, the image to be tested f (size n × m) is shrunk horizontally, and its height is kept unchanged. The shrunken image $T^{\prime }$ is obtained.

(7) $$T^{\prime }(i,j^{\prime })=\frac{\sum_{j=\left\lfloor sk\;\times \;(j^{\prime }-1)\right\rfloor +1}^{\left\lfloor sk\;\times \;j^{\prime }\right\rfloor }f(i,j)}{sk}$$

where $T^{\prime }$ is the shrunken image in the horizontal direction; i is the row coordinate of $T^{\prime }$ which takes a value in the range of 1,2,…,n; $j^{\prime }$ is the column coordinate of $T^{\prime }$, which takes values in the range of $1,2,\ldots ,\left\lfloor m/sk\right\rfloor$; j is the column coordinate of f, which takes values in the range 1,2,…,m; ⌊ ⌋ is a downward rounding symbol; and sk is the scale factor, sk = (columns of the original image)/(columns of the target image). To better explain the concept of algorithm development for horizontal shrink, we introduce the sk variable in place of the w variable. The two variables have different physical meanings but can be considered numerically identical.

After the operation of shrinking, the abnormal column in the original image is diluted by the data of the normal column, at which point it can be approximated that there is no abnormal data in the shrunken image $T^{\prime }$. To ensure size consistency between the template and the expanded image f, this paper adopts the bilinear interpolation method to enlarge $T^{\prime }$ to the size of n × m to obtain the final self-referencing template T.

Calculate the absolute residual of the self-referencing template T and the expanded image f to obtain the residual map Res.

(8) Res(x,y)=abs(T(x,y)−f(x,y))

where abs is the absolute value operation.

By choosing different values of sk, a series of self-referencing algorithm residual maps of different scales can be obtained. Since different sk values have different anomaly detection effects for different sizes of anomalous regions, it can be assumed that the residual map corresponding to a series of sk values must contain all the anomalous regions. The mean value of these residual maps can be used to extract all the anomalous regions; this step is mainly to find all the anomalous regions without requiring all the anomalous regions to have a high response. The mean value of all residual maps is calculated to obtain the multi-scale self-referencing anomaly detection score $Score_{ms}$.

(9) $$Score_{ms}=\frac{\sum_{k=1}^{Num}Res_{k}}{Num}$$

where k is the index number of the scale factor k=1,2,⋯,Num; the scale factor takes values in the array $\left[sk_{1},sk_{2},\cdots sk_{Num}\right]$; and $Res_{k}$ is the residual map obtained when the scale factor $sk=sk_{k}$.

To perform the normalization operation on $Score_{ms}$, introduce the normalization threshold $t_{1}$.

(10) $$Score_{ms}=\left\{\begin{array}{cc} \begin{array}{c} Score_{ms}/\max(Score_{ms})\\ Score_{ms}/t_{1} \end{array} & \begin{array}{c} \max(Score_{ms})\geq t_{1}\\ \max(Score_{ms})<t_{1} \end{array} \end{array}\right.$$

where $\max(Score_{ms})$ is the maximum value of $Score_{ms}$.

At this time, $Score_{ms}$ contains not only the response of the anomalous region but also the response of the non-anomalous region, but there is less phase interference in it, and the information of the mean grayscale template MGT can be introduced to suppress the non-anomalous region. Since the a priori anomaly score $Score_{prior}$ has a high response to both the significant anomaly region and the phase disturbance, the a priori anomaly scores $Score_{prior}$ are operated with the multi-scale self-referencing anomaly scores $Score_{ms}$ for the dot product, and only the significant anomaly regions can be retained to obtain the scores of the significant anomaly regions $Score_{S}$.

(11) $$Score_{S}=Score_{prior}.\;\times \;Score_{ms}$$

The parameter $t_{2}$ is introduced for contrast enhancement of $Score_{S}$.

(12) $$Score_{S}=\{\begin{array}{cc} \begin{array}{c} 1\\ Score_{S}/t_{2} \end{array} & \begin{array}{c} \max(Score_{S})\geq t_{2}\\ \max(Score_{S})<t_{2} \end{array} \end{array}$$

At this time, the interference caused by phase anomalies and large-size anomalies is removed from the $Score_{S}$, but it is less effective in detecting the anomalous regions that are close to the grayscale of MGT. Here, the strategies of image restoration and, again, multi-scale self-referencing templates are introduced to solve the above problems. The main idea is to fill the anomalous position in the original image f with the mean grayscale template MGT corresponding position data according to the significant anomaly detection score $Score_{S}$.

The dot product operation is performed on $Score_{S}$ and MGT to obtain the grayscale data $MGT^{\prime }$ for restoration.

(13) $$MGT^{\prime }=MGT.\;\times \;Score_{S}$$

The locations to be reconstructed in f are removed with $Score_{S}$ to obtain the anomaly region mask map $f_{mask}$.

(14) $$f_{mask}=f.\;\times \;(1-Score_{S})$$

The final restoration map R is obtained by adding $MGT^{\prime }$ and $f_{mask}$.

(15) $$R=MGT^{\prime }+f_{mask}$$

The final anomalous region score result $Score_{final}$ is obtained by using the multi-scale self-referencing template method in Equations (7)–(9) again for the restoration map R. In $Score_{final}$, phase errors are eliminated, the effect of large size anomalies is overcome and the response to low-contrast anomalies is high.

Since the response of the anomalous region is usually not uniform, in order to achieve the complete segmentation of the anomalous region, the region growth method is introduced here, setting the region growth start point $t_{3}$ and the region growth end point $t_{4}$. The final anomalous region segmentation result Result can be obtained.

## 4. Experiment

### 4.1. Bilinear Interpolation Unfolding Effect

**Figure 8:**
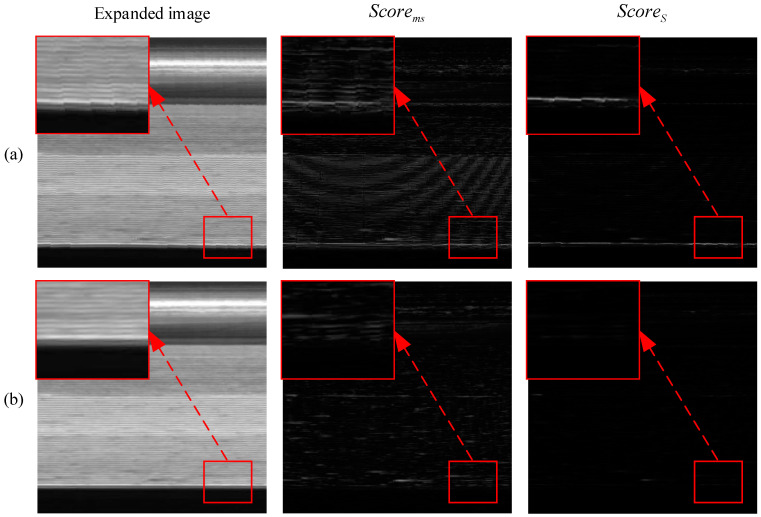
Bilinear interpolation unfolding effect. (**a**) Nearest-neighbor interpolation. (**b**) Bilinear interpolation.

Image unfolding is a key part of the data processing of the algorithm in this paper, where the thin-walled annular region of interest needs to be unfolded into a long strip of images to facilitate the development of the algorithm. The region of interest of the ring is converted to a rectangular region, and the area of the region of interest increases so it involves interpolation of the pixels of the increased region in the unfolded image. Since the algorithm of this paper is based on the uniformity of grayscale in the horizontal direction of the unfolded image, if the interpolation method is not well chosen, it leads to the generation of a jagged texture in the horizontal direction of the unfolded image, as shown in Figure 8a, which affects the detection effect of the algorithm of this paper.

Figure 8 demonstrates the image unfolding results of the nearest-neighbor and bilinear interpolation methods and shows $Score_{ms}$ (the multi-scale self-referencing anomaly detection score) and $Score_{S}$ (the significant anomaly detection score) processed by this paper’s algorithm. As shown in Figure 8, the generation of jagged edges in the expanded image can be avoided by the bilinear interpolation method, and, for the algorithm in this paper, the anomalous response interference from jagged edges can also be effectively suppressed.

As shown in the comparison results, the use of the bilinear interpolation method for image unfolding can effectively increase the degree of smoothness of the unfolding map to avoid the generation of jagged edges, which ensures the normal operation of the anomaly detection algorithm in this paper.

### 4.2. Multi-Scale Parameter Determination

**Figure 9:**
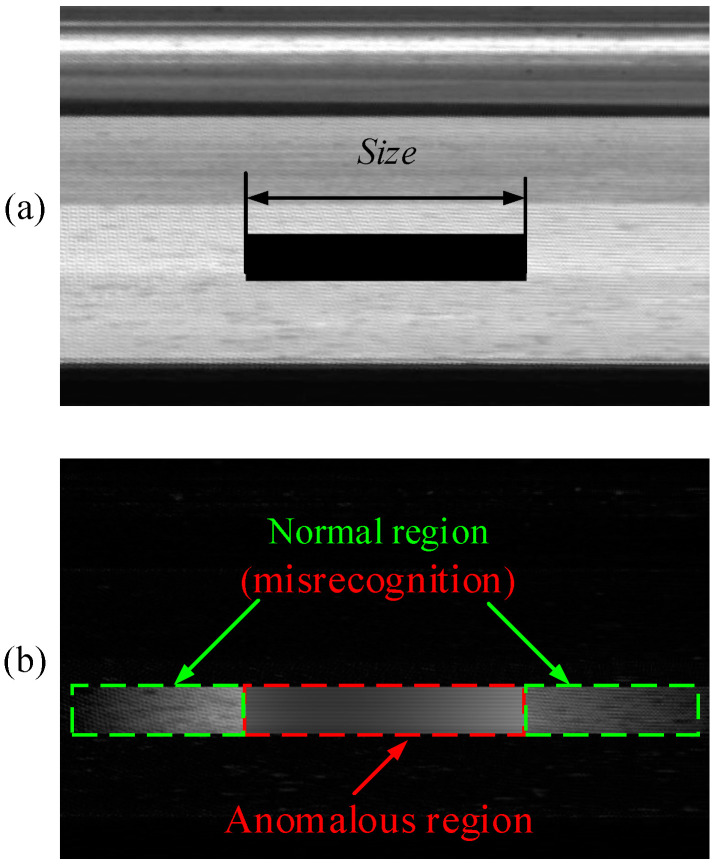
Artificial anomalous region and the residual maps Res. (**a**) Artificial anomalous regions. (**b**) Self-referencing template residual map Res.

The scale factor sk is related to the horizontal dimension of the anomalous region to be detected in the expanded image. When the value of sk is too small, the algorithm becomes weak in detecting anomalies of large size, and, at the same time, it causes misrecognition, as in the green area of Figure 9b. If sk is too large, it introduces phase or light uneven interference. Under actual working conditions, the size of the anomaly region varies greatly, and here in this paper, the pixel range of the horizontal size of the anomalous region is defined as 5~500 pixels. As shown in Figure 9a, in this paper, the self-referencing residual map Res is calculated for different sizes of artificial anomalous regions with different sk values, and the mean response value v of the anomalous region (red region in Figure 9b) is counted to determine the choice of the scale factor sk.

**Figure 10:**
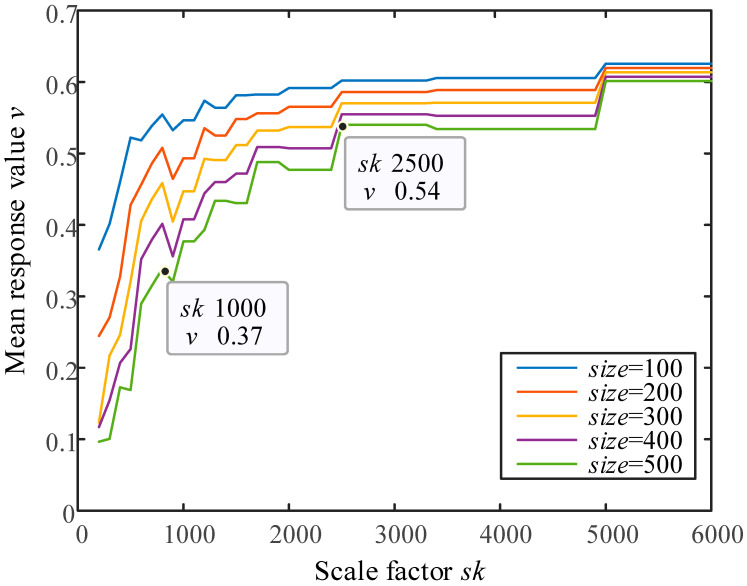
Experimental statistical graph of scale factor sk.

The statistical results of the scale factor sk versus the mean response value v for the anomalous region are shown in Figure 10. The mean response value v of the anomalous region increases with the scale factor sk. As can be seen from the size of the anomalous region, the scale factor sk corresponding to the convergence position of the average response value increases as the size increases, while the final convergence value becomes smaller, which is consistent with the calculation principle of the self-referencing template method. From the figure, it can be seen that when size takes the maximum value of 500, the mean response value v takes the basic convergence at sk=2500. Since the residual response is a grayscale difference, and considering that the human eye has the highest discrimination ability for grayscale display systems with a grayscale level of 8, that is, in the residual map Res, from 0 to 1, when the residual value > 1/8 = 0.125, the corresponding position can be considered as an anomalous region. To avoid the interference caused by the large-scale factor and to ensure the effect of anomaly detection, this paper takes sk=1000 when the mean response value v is 0.37, which meets the reliability standard of anomaly detection.

The grayscale value of the artificial anomalous region in this paper is 0, which does not represent the full true anomaly region. However, due to the high error tolerance of the chosen average response value v, combined with a large number of supplementary experiments, this paper suggests that the algorithm in this paper has a better anomaly detection effect when the scale parameter sk is taken to be twice the maximum size of the anomalous region to be detected.

In summary, for the anomaly detection demand of 5~500 pixels in a horizontal direction, the scale factor sk selected in this paper takes the value range of 10~1000, and, to ensure that sk is an integer, the number of sk is taken as 6 by equal difference interval, and sk=10,208,406,802,1000.

### 4.3. Determination of Algorithm Grayscale Parameters

There are four grayscale parameters in this algorithm, which are the normalization threshold $t_{1}$ for normalizing the multi-scale self-referencing anomaly detection score $Score_{ms}$, the threshold $t_{2}$ for contrast enhancement of the significant anomaly detection score $Score_{S}$, the anomalous region growth start point $t_{3}$ and end point $t_{4}$.

The normalization threshold $t_{1}$ serves to prevent the multi-scale self-referencing template score $Score_{ms}$ from being too low, which, in turn, amplifies the phase and noise disturbances during normalization. Studies have shown that the human eye has a high-resolution accuracy for regions with grayscale differences >0.125, i.e., the human eye resolution threshold is 0.125. Here, $t_{1}$ is set to equal the human eye resolution threshold of 0.125. The main function of contrast enhancement threshold $t_{2}$ for the significant anomalous region is to improve the contrast of anomalous regions for subsequent image restoration. If its value is too large, it affects the low-contrast abnormality recognition; if its value is too small, it introduces too much noise interference. To ensure the detection effect, its value is also taken to be the human eye resolution threshold 0.125.

**Figure 11:**
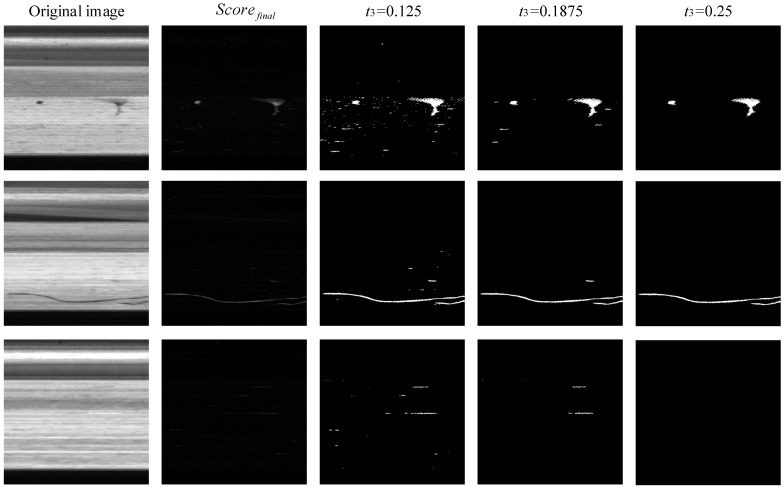
The region growth start point $t_{3}$ comparison experiment.

The region growth parameters $t_{3}$ and $t_{4}$ are the key parameters that control the final segmentation results of the algorithm. Figure 11 shows the region growth start point $t_{3}$ comparison experiment. The starting value is taken as the human eye resolution threshold 0.125 for $t_{3}$ = 0.125, 0.1875 and 0.25, at which $t_{4}$ = 0.1. From the results of the experiment, it can be seen that if $t_{3}$ is too low, too much interference is introduced, and the noise is significantly suppressed with the increase in $t_{3}$. Therefore, this paper selects the starting point of regional growth as $t_{3}$ = 0.25.

**Figure 12:**
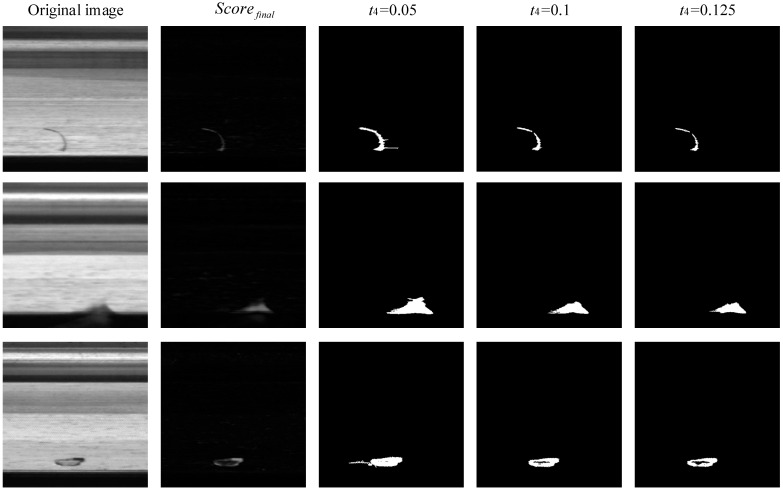
The region growth end point $t_{4}$ comparison experiment.

From Figure 12, it can be seen that if the region growth end point $t_{4}$ is too low, too much noise interference is introduced, and, as $t_{4}$ increases, the noise interference decreases but the integrity of the segmentation is reduced. Considering the suppression of noise interference and the integrity of the abnormal region segmentation, this paper selects the region growth end point as $t_{4}=0.1$, whose value is slightly lower than the human eye resolution threshold.

### 4.4. Effect of the a Priori Information on Detection

The self-referencing template method is less effective in detecting large-size anomalous regions but has a good suppression effect on phase errors, while the mean grayscale template is more effective in detecting large-size anomalies but introduces excessive phase errors. To verify the detection effect of the method in this paper for large-size anomalies and the suppression effect of phase errors, three different samples of large defects in the annular metal turning surface are selected in this section to compare the detection effect of the self-referenced template, mean grayscale template and the method in this paper.

**Figure 13:**
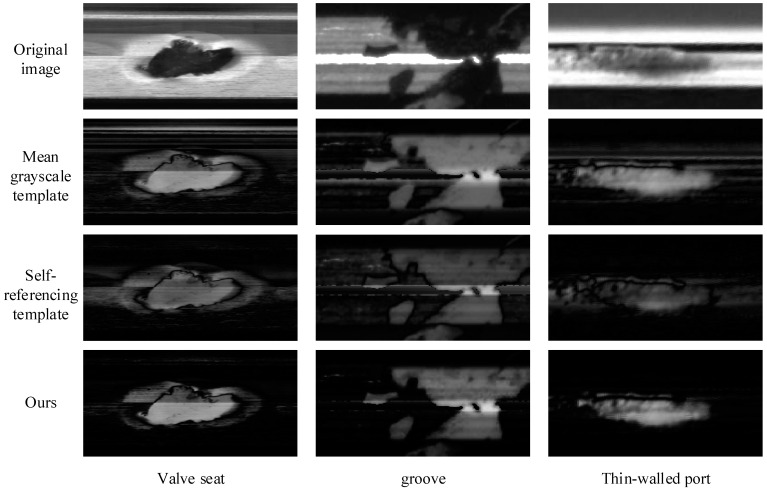
Comparison experiment of large-size anomaly detection effect.

Figure 13 shows the comparison experiment of large-size anomaly detection effect. The figure shows that the direct use of the mean grayscale template works well for detecting large-size anomalous regions, but phase interference is introduced; self-referencing methods are effective in suppressing phase interference but have a low response for large sizes, while also introducing erroneous responses in non-anomalous regions; the a-priori-information-based and multi-scale self-referencing template algorithm proposed in this paper combines the advantages of the mean grayscale template and self-referencing template to ensure the detection of large-size anomalies while effectively suppressing phase interference.

### 4.5. Comparison of Commonly Used Unsupervised Anomaly Detection Methods

The algorithm in this paper is tested against three commonly used unsupervised anomaly detection algorithms, CFLOW-AD, PatchCore and PaDiM, which perform well in the MVTec AD dataset. Since the object processed by the algorithm in this paper is a long expanded image, and the commonly used unsupervised anomaly detection algorithms CFLOW-AD, PatchCore and PaDiM process images that are square, to compare the performance of this paper with the above commonly used methods, in this part of the experiment, this paper intercepts the expanded images as samples of equal length and width to construct the comparison experimental dataset, as shown in Table 2.

The programming environment of the algorithm in this paper is MatlabR2021b, and the programming environment of the comparison algorithm is Python, and the experiments are conducted in a Windows 10 system. The slave computer is configured with an Intel(R) Core (TM) i5-11400F @ 2.60 GHz 2.59 GHz and a memory of 16 G. The model of graphics card is an NVIDIA GeForce RTX 3060 with 12 GB memory.

Two evaluation indicators, AUROC and IOU, are selected. AUROC is a common indicator in the field of anomaly detection and is used to evaluate the anomaly response of each algorithm, which avoids the influence of subjective assumptions in threshold selection by summarizing the overall performance of the model under all possible thresholds. The IOU indicator is used to evaluate the anomalous region segmentation capability of each algorithm.

As can be seen from the Table 3, in terms of the Image AUROC and Pixel AUROC indicators, this paper’s method achieves the optimal mean indicator for all three data. In terms of the Image AUROC indicator, the advantage of this method is more obvious compared with that of other methods. The reason for this is that this method has a higher response score for anomalous regions based on the grayscale difference, but the response score is not uniform over the same anomalous region. As a result, compared with other methods, this paper is better in terms of the Image AUROC indicator but slightly stronger in the Pixel AUROC indicator.

The goal of anomaly detection is to segment the anomalous region, and the AUROC indicator more greatly reflects the divisibility of the anomalous region response score obtained by the algorithm. The ease of partitioning of the anomalous region response scores also needs to be considered when performing algorithm performance evaluation. In this section, we compare the image-level mean IOU indicator M_IOU of the anomalous region segmentation results of the method in this paper with those of other classical unsupervised methods, and the experimental results are shown in Table 4.

From the M_IOU indicator values in the table, it can be seen that the M_IOU of all algorithms exceeds 0.5, indicating that the algorithms all have excellent anomaly segmentation ability. Compared with the Pixel AUROC indicator, the advantage of this paper’s method is more obvious in the M_IOU indicator. The reason is that the Pixel AUROC indicator is affected by the area of the anomalous region; if the large anomalous region is correctly located, the Pixel AUROC indicator is greatly improved, while if the small anomalous region is incorrectly located, there is only a small decrease in the Pixel AUROC indicator. However, when the IOU is counted from each image, and the mean value is calculated, the effect of anomalous region size on the indicator is effectively overcome. The experimental results further demonstrate the effectiveness of the method in this paper.

**Figure 14:**
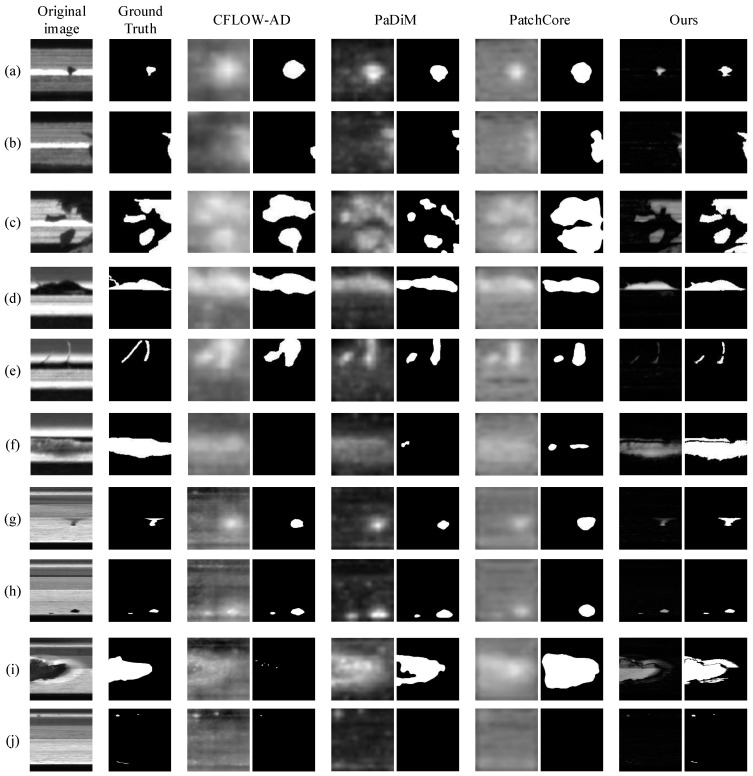
Comparison results of anomaly detection algorithms. (**a**–**c**) are grooves data. (**d**–**f**) are thin-walled port data. (**g**–**j**) are valve seat data. Each column in the figure corresponds to the results processed by the original image, Ground Truth, and 4 detection methods, with anomalous response results and final segmentation results shown under each detection method.

Some of the experimental results are shown in Figure 14. Comparing the anomalous response results of the algorithms, the edges of the anomalous response region of the commonly used unsupervised anomaly detection methods are fuzzy, and the anomalous response region of this paper’s method is more precise, responding only to the anomalous region and not to the non-anomalous region. From the segmentation results in the figure, it can be seen that the anomaly detection method proposed in this paper achieves the optimal anomaly localization results in contrast to Ground Truth. As shown in Figure 14b,g, the method in this paper still realizes the complete segmentation of defects in the case of an inhomogeneous anomalous response due to the segmentation strategy of region growing. For large-size anomalous regions, as shown in Figure 14c,f,i, the response of this paper’s method to the anomalous regions is more significant compared with that of other comparison methods, and it avoids the problem of the response of the anomalous regions not being significant. For small-size anomalous regions, as shown in Figure 14j, the method in this paper still achieves good detection results based on the grayscale difference, while the commonly used anomaly detection methods have a very low response (CFLOW-AD and PaDiM) or no response (PatchCore).

In this paper, the operational efficiency of each algorithm is also analyzed. As shown in Table 5, the image sizes processed by each algorithm differ slightly when speed tests are performed due to the different input data sizes required by the different algorithms. In terms of efficiency, the algorithm in this paper achieves the highest detection speed.

The experimental results in this section demonstrate that the algorithm in this paper is suitable for annular metal turning surfaces with both good anomalous region segmentation capability and high speed, while the algorithm in this paper has no excessive configuration requirements for processing units and is easy to deploy for applications.

## 5. Conclusions

This paper focuses on solving the problem of anomaly detection in annular metal turning surface in industrial application scenarios. In this paper, the imaging characteristics of annular metal turning surfaces are utilized to unfold the annular region of interest into a rectangular region, which reduces the amount of data processing and simplifies the development of subsequent algorithms. This paper introduces statistical grayscale a priori information, which effectively improves the detection of large-size anomalous regions. Through the acquisition of self-referencing multi-scale templates, combining the image’s own information with the a priori information, the interference caused by contours and large-size anomalous regions is effectively suppressed. The segmentation operation of the response map using the region growing method improves the completeness of anomalous region segmentation. Compared to existing unsupervised anomaly detection algorithms, the method in this paper achieves the best average detection results in the three datasets of groove, thin-walled ports and valve seat data. The experimental results show that this method achieves a mean Pixel AUROC of 0.977, and the mean M_IOU of segmentation reaches 0.788. In terms of efficiency, this method is also much more efficient than the commonly used anomaly detection algorithms. The method proposed in this paper is versatile for annular metal turning surfaces and has lower hardware requirements and superior economics for industrial applications.

It is worth noting that the anomaly detection algorithm proposed in this paper is mainly for annular turning surfaces with uniform grayscale, and the algorithm is not applicable to objects with uneven grayscale in the expanded image, such as flange surfaces with holes and gear end faces. The algorithm in this paper still relies on grayscale differences and currently performs poorly for textural anomalies with very low grayscale differences (e.g., rippling). In future research, we intend to incorporate texture information into the development of the algorithm, as well to improve the detection of texture anomalies, and we will also explore the optimization of the algorithm in this paper to accommodate objects to be detected with uneven grayscale in the horizontal direction.

## Figures and Tables

**Table 1:** Composition of the annular metal turning surface dataset.

Dataset	Number of Positive Samples	Number of Negative Samples	Expanded Image Size (Pixels)
Groove	98	29	100 × 7000
Thin-walled port	89	34	120 × 8700
Valve seat	128	32	440 × 10,000

**Table 2:** Comparison experimental dataset.

Data	Size (Pixels)	Number of Positive-Sample Training Set	Number of Test Set	
			Positive Sample	Negative Sample
Groove	100 × 100	204	29	29
Thin-walled port	120 × 120	211	34	34
Valve seat	440 × 440	237	32	32

**Table 3:** **AUROC** indicator performance comparison.

Item	Algorithm	Image AUROC			Mean	Pixel AUROC			Mean
		Groove	Thin-Walled Port	Valve Seat		Groove	Thin-Walled Port	Valve Seat	
1	CFLOW-AD	0.981	**0.971**	0.901	0.951	0.941	0.973	0.904	0.939
2	PaDiM	0.977	0.930	0.934	0.947	0.968	**0.975**	0.982	0.975
3	PatchCore	**1**	0.958	0.945	0.968	0.960	0.965	**0.986**	0.970
4	Ours	**1**	0.959	**1**	**0.986**	**0.976**	**0.975**	0.981	**0.977**

**Table 4:** **M_IOU** indicator performance comparison.

Item	Algorithm	M_IOU			Mean
		Groove	Thin-Walled Port	Valve Seat	
1	CFLOW-AD	0.573	0.595	0.52	0.563
2	PaDiM	0.582	0.567	0.575	0.575
3	PatchCore	0.66	0.617	0.584	0.620
4	Ours (score_s_)	**0.843**	**0.691**	**0.831**	**0.788**

**Table 5:** Algorithm efficiency comparison.

Algorithm	Data	Input Size	FPS
CFLOW-AD	Groove	100 × 100	28.71
	Thin-walled port	120 × 120	22.35
	Valve seat	440 × 440	4.56
PaDiM	Groove	224 × 224	1.72
	Thin-walled port	224 × 224	1.58
	Valve seat	224 × 224	1.89
PatchCore	Groove	224 × 224	0.55
	Thin-walled port	224 × 224	0.78
	Valve seat	224 × 224	0.86
Ours	Groove	100 × 100	411.76
	Thin-walled port	120 × 120	276.51
	Valve seat	440 × 440	17.73

## Data Availability

Not applicable.

## References

[1] Dongling Y., Xiaohui Z., Jianzhen Z., Nanxing W. (2022). An enhancement algorithm based on adaptive updating template with Gaussian model for Si_3_N_4_ ceramic bearing roller surface defects detection. Ceram. Int..

[2] Cheng S., Lu J., Yang M., Zhang S., Xu Y., Zhang D., Wang H. (2023). Wheel hub defect detection based on the DS-Cascade RCNN. Measurement.

[3] Zhao Y., An X., Sun N. (2020). Virtual simulation experiment of the design and manufacture of a beer bottle-defect detection system. Virtual Real. Intell. Hardw..

[4] Suo X., Liu J., Dong L., Shengfeng C., Enhui L., Ning C. (2022). A machine vision-based defect detection system for nuclear-fuel rod groove. J. Intell. Manuf..

[5] Chen Y., Li Y., Zhang H., Tong L., Cao Y. (2016). Automatic power line extraction from high resolution remote sensing imagery based on an improved Radon transform. Pattern Recognit..

[6] He Z., Sun L. (2015). Surface defect detection method for glass substrate using improved Otsu segmentation. Appl. Opt..

[7] Chen J., Liu Z., Wang H., Núñez A., Han Z. (2017). Automatic Defect Detection of Fasteners on the Catenary Support Device Using Deep Convolutional Neural Network. IEEE Trans. Instrum. Meas..

[8] Xiang J., Wang J., Zhou J., Meng S., Pan R., Gao W. (2021). Fabric defect detection based on a deep convolutional neural network using a two-stage strategy. Text. Res. J..

[9] Alipour M., Harris D.K., Miller G.R. (2019). Robust Pixel-Level Crack Detection Using Deep Fully Convolutional Neural Networks. J. Comput. Civ. Eng..

[10] Zhao S., Chen H., Wang C., Shi S. (2023). SNCF-Net: Scale-aware neighborhood correlation feature network for hotspot defect detection of photovoltaic farms. Measurement.

[11] Cha Y.-J., Choi W., Büyüköztürk O. (2017). Deep Learning-Based Crack Damage Detection Using Convolutional Neural Networks. Comput. Aided Civ. Infrastruct. Eng..

[12] Atha D.J., Jahanshahi M.R. (2018). Evaluation of deep learning approaches based on convolutional neural networks for corrosion detection. Struct. Health Monit..

[13] Wang T., Chen Y., Qiao M., Snoussi H. (2017). A fast and robust convolutional neural network-based defect detection model in product quality control. Int. J. Adv. Manuf. Technol..

[14] Deng S., Cai W., Xu Q., Bo L. Defect detection of bearing surfaces based on machine vision technique. Proceedings of the International Conference on Computer Application & System Modeling.

[15] Xiaoxun Z., Xinyu H., Xiaoxia G., Xing Y., Zixu X., Yu W., Huaxin L. (2022). Research on crack detection method of wind turbine blade based on a deep learning method. Appl. Energy.

[16] Lu Q., Lin J., Luo L., Zhang Y., Zhu W. (2022). A supervised approach for automated surface defect detection in ceramic tile quality control. Adv. Eng. Inform..

[17] Zhou Z., Yan L., Zhang J., Zheng Y., Gong C., Yang H., Deng E. (2023). Automatic segmentation of tunnel lining defects based on multiscale attention and context information enhancement. Constr. Build. Mater..

[18] Zhang H., Li H., Chen N., Chen S., Liu J. (2022). Novel fuzzy clustering algorithm with variable multi-pixel fitting spatial information for image segmentation. Pattern Recognit..

[19] Tsai D.-M., Rivera Molina D.E. (2019). Morphology-based defect detection in machined surfaces with circular tool-mark patterns. Measurement.

[20] Wang K.F. (2019). Quantitative detection of internal defects based on morphological opening, filling and binarizing operations on wrapped phase of out-of-plane deformation in digital speckle pattern interferometry. NDT E Int..

[21] Kong Q., Wu Z., Song Y. (2022). Online detection of external thread surface defects based on an improved template matching algorithm. Measurement.

[22] Vaikundam S., Hung T.Y., Liang T.C. Anomaly region detection and localization in metal surface inspection. Proceedings of the 2016 IEEE International Conference on Image Processing (ICIP).

[23] Tao X., Zhang D., Ma W., Hou Z., Lu Z., Adak C. (2022). Unsupervised Anomaly Detection for Surface Defects with Dual-Siamese Network. IEEE Trans. Ind. Inform..

[24] Collin A.S., Vleeschouwer C.D. Improved anomaly detection by training an autoencoder with skip connections on images corrupted with Stain-shaped noise. Proceedings of the 2020 25th International Conference on Pattern Recognition (ICPR).

[25] Schlegl T., Seeböck P., Waldstein S.M., Schmidt-Erfurth U., Langs G., Niethammer M., Styner M., Aylward S., Zhu H., Oguz I., Yap P.-T., Shen D. (2017). Unsupervised Anomaly Detection with Generative Adversarial Networks to Guide Marker Discovery.

[26] Gudovskiy D., Ishizaka S., Kozuka K. CFLOW-AD: Real-Time Unsupervised Anomaly Detection with Localization via Conditional Normalizing Flows. Proceedings of the IEEE/CVF Winter Conference on Applications of Computer Vision.

[27] Salehi M., Sadjadi N., Baselizadeh S., Rohban M.H., Rabiee H.R. (2020). Multiresolution Knowledge Distillation for Anomaly Detection. arXiv.

[28] Bergmann P., Batzner K., Fauser M., Sattlegger D., Steger C. (2021). The MVTec Anomaly Detection Dataset: A Comprehensive Real-World Dataset for Unsupervised Anomaly Detection. Int. J. Comput. Vis..

[29] Defard T., Setkov A., Loesch A., Audigier R. PaDiM: A Patch Distribution Modeling Framework for Anomaly Detection and Localization. Proceedings of the Pattern Recognition, ICPR International Workshops and Challenges.

[30] Roth K., Pemula L., Zepeda J., Schlkopf B., Gehler P. Towards Total Recall in Industrial Anomaly Detection. Proceedings of the 2022 IEEE/CVF Conference on Computer Vision and Pattern Recognition (CVPR).

[31] Yun J.P., Choi S.H., Seo B., Sang W.K. (2008). Real-time vision-based defect inspection for high-speed steel products. Opt. Eng..

[32] Tsai D.M., Lai S.C. (2008). Defect detection in periodically patterned surfaces using independent component analysis. Pattern Recognit..

[33] Jing J.-F., Chen S., Li P.-F. (2017). Fabric defect detection based on golden image subtraction. Color. Technol..

